# Tobacco Smoke Exposure and Levels of Urinary Metals in the U.S. Youth and Adult Population: The National Health and Nutrition Examination Survey (NHANES) 1999–2004

**DOI:** 10.3390/ijerph6071930

**Published:** 2009-07-02

**Authors:** Patricia A. Richter, Ellen E. Bishop, Jiantong Wang, Monica H. Swahn

**Affiliations:** 1 Office of Smoking and Health, Centers for Disease Control and Prevention, Atlanta, GA 30341, USA; 2 Chronic & Infectious Disease Research Program, RTI International. Atlanta, GA 30341, USA; E-Mails: ebishop@rti.org (E.B.); wang@rti.org (J.W.); 3 Institute of Public Health, College of Health and Human Sciences, Georgia State University, Atlanta, GA 30302, USA; E-Mail: Mswahn@gsu.edu

**Keywords:** secondhand smoke (SHS), metals, youth, lead, cadmium, race/ethnicity, tobacco smoke, smoker, toxicity

## Abstract

We assessed 12 urine metals in tobacco smoke-exposed and not exposed National Health and Nutrition Examination Survey participants. Our analysis included age, race/ethnicity, and poverty status. Gender and racial/ethnic differences in cadmium and lead and creatinine-adjusted and unadjusted data for group comparisons are presented. Smokers’ had higher cadmium, lead, antimony, and barium levels than nonsmokers. Highest lead levels were in the youngest subjects. Lead levels among adults with high second-hand smoke exposure equaled smokers. Older smokers had cadmium levels signaling the potential for cadmium-related toxicity. Given the potential toxicity of metals, our findings complement existing research on exposure to chemicals in tobacco smoke.

## 1. Introduction

Cigarette smoke inhaled by a smoker contains more than 4,000 chemicals [1], and second hand smoke (SHS) is qualitatively similar [1]. Metals in tobacco smoke are of public health concern because of their potential toxicity and carcinogenicity [2]. Some are linked to adverse health outcomes such as cardiovascular and renal disease and impaired lung function among smokers [3–7]. Because the dose of toxic chemicals received by a smoker is an important factor in the harm caused by tobacco, analysis of the National Health and Nutrition Examination Survey (NHANES) exposure data suggests promising candidate chemicals for determining whether reductions of certain toxic constituents in cigarettes and cigarette smoke translate into reductions in active and passive smokers’ exposure to these substances.

Prevention of tobacco use and SHS exposure remains a critically important public health priority. The Surgeon General recently concluded that there is no safe level of SHS exposure [1,8]. Although significant progress has been made to reduce SHS exposure, nearly half (46.4%) of U.S. nonsmokers remain exposed [9].

The NHANES is a vital source of population-level exposure data for environmental pollutants, including tobacco smoke. Population-level data is important to assess the magnitude of various exposures and the impact of laws and policies aimed at tobacco use and SHS exposure [2,10]. All 12 of the metals in this study (antimony (Sb), barium (Ba), beryllium (Be), cadmium (Cd), cesium (Cs), cobalt (Co), lead (Pb), mercury (Hg), molybdenum (Mo), platinum (Pt), thallium (Tl), and tungsten (W)) have been reported in tobacco smoke [11,12]. Some metals, such as cadmium and lead, are widely recognized for their toxicity and tendency to accumulate in the body [13,14]. While noting that no one threshold for all adverse effects from lead exposure has been demonstrated, and that the available evidence has important limitations including the small number of studies of peak blood lead levels below 10 μg/dL in children never known to have a blood lead level exceeding 10 μg/dL, the CDC has concluded that the data demonstrates that no level of lead in a child’s blood can be specified as safe [15,16]. Other metals, such as cobalt, have beneficial or harmful effects, depending on the level of exposure [17].

This study examined levels of urinary metals among cigarette smoke exposed and not exposed NHANES participants. Unlike previous studies that focused on a select few metals [5,6], we included 12 metals to provide population-level, baseline exposure data for a large number of potentially toxic metals. We analyzed urine data because, while influenced by pharmacokinetics, urine measurements are a useful noninvasive approach in biomonitoring research of exposures to metals and other environmental pollutants [18–21]. The findings from this study contribute to the limited information available regarding exposure to potentially toxic metals in tobacco smoke. By analyzing data for both active and passive smokers, we provide information to strengthen public health messages regarding quitting smoking, to increase awareness of the dangers of SHS exposure, and the need for strategies to further reduce SHS exposure.

## 2. Results and Discussion

The unweighted sample by demographic characteristic is presented for all metals (Table 1). Approximately equal numbers of males and females are present among participants and most were above the poverty threshold.

Table 2 shows the creatinine adjusted and unadjusted geometric mean levels for the overall population and the four exposure groups (unexposed nonsmokers (nonsmokers); nonsmokers with low SHS exposure (nonsmokers_low_); nonsmokers with high SHS exposure (nonsmokers_high_); and self-identified cigarette smokers (smokers)).

Nonsmokers_low_ had significantly higher lead (adjusted; p < 0.05 and unadjusted; p < 0.001) and antimony (unadjusted; p < 0.01) levels than nonsmokers. There was no significant difference in levels of urine cadmium, barium, cesium, cobalt, molybdenum, platinum, thallium, or tungsten for nonsmokers_low_ and nonsmokers (Table 2).

Smokers had higher cadmium, lead (p < 0.001), antimony (unadjusted; p < 0.001), and barium (unadjusted; p < 0.01) levels than nonsmokers. Levels of some metals, including essential metals cobalt (adjusted; p < 0.05) and molybdenum (p < 0.05), were lower in smokers (Table 2).

Table 3 presents adjusted and unadjusted geometric mean cadmium levels for exposure groups by age, race/ethnicity, gender, and poverty status. Cadmium levels generally increased with age in the overall population and in each exposure group. Except for 65 and older nonsmokers_high_ that had a higher urine cadmium than nonsmokers_low_ (adjusted; p < 0.01), nonsmokers did not have an increase in urine cadmium across the exposure groups. Smokers 19–35 (adjusted; p < 0.05 and unadjusted; p < 0.001), 35–50 (p < 0.001), 50–65 (p < 0.001), and 65 and older (p < 0.001) had higher levels than did similarly aged nonsmokers. Levels in smokers 35–50, 50–65, and 65 years and older were also higher than levels in nonsmokers_high_ (p < 0.001). Smokers in all racial/ethnic groups had higher levels than nonsmokers (p < 0.001).

Analysis of unadjusted data show that, except for nonsmokers_low_, non-Hispanic blacks had higher cadmium levels than non-Hispanic whites (nonsmokers, p < 0.01; nonsmoker_high_, p < 0.01; smokers, p < 0.001). When means were creatinine adjusted, non-Hispanic blacks had lower or similar levels than non-Hispanic whites. Mexican Americans overall (adjusted; p < 0.001 and unadjusted; p < 0.05), Mexican American nonsmokers (adjusted; p < 0.001 and unadjusted; p < 0.05), and Mexican American nonsmokers_low_ (adjusted; p < 0.01) had lower urine cadmium levels than non-Hispanic whites.

Male and female smokers had higher adjusted and unadjusted cadmium levels than nonsmokers (p < 0.001). Male and female nonsmokers did not have an increase in urine cadmium across the exposure groups. Females (all exposure groups) had higher adjusted levels than males (p < 0.001).

Levels of cadmium in the urine of smokers above and below the poverty threshold were higher than levels of nonsmokers (p < 0.001). Nonsmokers above and below the poverty threshold did not have an increase in urine cadmium across the exposure groups.

Table 4 presents adjusted and unadjusted geometric mean lead levels for all exposure groups by age, race/ethnicity, gender, and poverty status. Among 6–12 year olds, nonsmokers_high_ had higher levels than nonsmokers (adjusted; p < 0.001 and unadjusted; p < 0.05) or nonsmokers_low_ (adjusted; p < 0.01). Excluding youth 6–12 years, levels generally increased with age in the overall population and for all exposure groups. Although 12–19 year old nonsmokers_low_ (adjusted; p < 0.05 and unadjusted; p < 0.01) had higher urine lead levels than 12–19 year old nonsmokers, most age categories did not have an increase in urine lead across the exposure groups. Smokers 12–19 (unadjusted; p < 0.05), 19–35 (p < 0.001), 35–50 (p < 0.001), 50–65 (p < 0.001), and 65 years and older (adjusted; p < 0.05 and unadjusted; p < 0.001) had significantly higher levels than did nonsmokers.

Both adjusted and unadjusted lead levels were higher in non-Hispanic white (adjusted; p < 0.01 and unadjusted p < 0.001) and non-Hispanic black smokers (p < 0.001) than in nonsmokers. Non-Hispanic black nonsmokers_high_ had higher levels than nonsmokers_low_ (adjusted; p < 0.001 and unadjusted; p < 0.05). Only the unadjusted level was higher in Mexican American smokers than nonsmokers (p < 0.001). Mexican American nonsmokers did not have an increase in urine lead across the exposure groups.

Across all levels of smoke exposure, non-Hispanic blacks (nonsmokers p < 0.001; nonsmokers_low_ p < 0.01; nonsmokers_high_ p < 0.001; smokers p < 0.001) and Mexican Americans (nonsmokers p < 0.001; nonsmokers_low_ p < 0.001; nonsmokers_high_ p < 0.01; smokers p < 0.001) had higher unadjusted lead levels than non-Hispanic whites. After adjusting for creatinine content, some differences remained among Mexican American nonsmokers (p < 0.001), nonsmokers_low_ (p < 0.001), and smokers (p < 0.05).

Adjusted and unadjusted lead levels in male smokers were higher than in male nonsmokers (p < 0.001). In female smokers, only the unadjusted level was higher than nonsmokers (p < 0.001). In general, unadjusted levels were significantly lower in females than males. Female nonsmokers, however, had significantly higher adjusted levels than male nonsmokers (p < 0.001).

When considering poverty status, adjusted and unadjusted lead levels of smokers above the poverty threshold were higher than levels in nonsmokers (p < 0.001). Nonsmokers_low_ above the poverty threshold had higher urine lead levels than nonsmokers above the poverty threshold (adjusted; p < 0.05 and unadjusted p < 0.01). For smokers below the poverty threshold, only the unadjusted urine lead level was higher than the level in nonsmokers (p < 0.05). Nonsmokers below the poverty threshold did not have an increase in urine lead across the exposure groups.

Our findings show that some metals are increased (e.g., cadmium and lead, and antimony and barium [unadjusted]) in smokers, while others are lower (e.g., mercury, beryllium, cesium, cobalt, molybdenum, platinum, and thallium [adjusted]) or unchanged (tungsten). In general, metals are absorbed in the lung, tend to persist in the body with half-lives of years to decades, and most are excreted through the kidneys and gastrointestinal tract [22]. So it is plausible that nontrivial exposures will be detectable in urine. Consequently, our findings of lower or unchanged urine levels suggest that, for the general population, cigarette smoke is not a major environmental source of exposure to mercury, beryllium, cesium, cobalt, molybdenum, platinum, thallium, or tungsten.

Lead and cadmium are emphasized because of their well established toxicity and tendency to accumulate in the body [13,14]. It is therefore particularly noteworthy that the highest lead levels were among nonsmokers_high_ 6–12 years, the youngest in our study population. It may be that the youth-adult disparity in SHS exposure, which has increased since the early 1990s [9], is contributing to children’s lead levels. This hypothesis is supported by others’ observations of high blood lead levels in children of smoking parents [3]. It has been suggested that smoking’s contribution to lead levels has become increasingly relevant as gasoline lead emissions have declined [3]. We found that, with few exceptions, adult nonsmokers_high_ and smokers had similarly high lead levels. Thus, while other potential sources of lead exposure were not assessed, our findings suggest that SHS exposure may be sufficient to produce a measurable, dose-dependent increase in lead levels. We also observed that only SHS-exposed nonsmokers above the poverty threshold had elevated urine lead. This suggests that tobacco smoke is an important source of lead exposure for those with higher household income and raises questions about other environmental exposures, across income and socioeconomic levels. Future studies of SHS-exposed children should be designed to address the possible contribution of early childhood environmental exposures to lead.

We also observed high barium and antimony in smokers compared to nonsmokers. As with cadmium and lead, barium and antimony are present in cigarette smoke [11,12]. Animal studies indicate that inhaled barium is primarily excreted in the feces [23]. In contrast, cadmium and absorbed lead are eliminated primarily in the urine [13,24]. Although only unadjusted barium was significantly elevated, the finding raises mechanistic considerations. For example, the amount of barium received by a smoker may exceed the normal renal capacity for tubular reabsorption through common competitive transcellular pathways [25] when exposure occurs in the presence of other divalent metals (e.g., cadmium). Kidney effects are the most sensitive endpoint following chronic barium exposure in laboratory animals [26] and one possible area of research is to investigate barium as a direct renal toxicant in smokers. Cadmium and lead are renal toxicants in humans and cadmium urine concentrations increase after kidney damage [25,27]. Renal tubular toxicity and low bone density are reported at urine cadmium levels as low as 1 μg/g [21,28]. Cadmium levels among older smokers in our study approach this level. Consequently, elevated urine barium among smokers may reflect decreased tubular reabsorption in a kidney damaged by exposure to cadmium or other metals. Antimony is excreted in the urine and feces [29]. Occupational and animal studies have reported respiratory and cardiovascular effects from inhalation of antimony compounds [29]. A recent study reported a sharp increase in risk of peripheral artery disease in individuals with low urine antimony levels below 0.1 μg/L and a persistent elevated risk above 0.1 μg/L compared with those with urine antimony at the limit of detection [7]. In our study, unadjusted urine antimony levels increased across exposure groups with smokers having the highest level of 0.13 μg/L. Oxidative stress is proposed to play a role in the toxicity and carcinogenicity of some metals (including cadmium, lead, and antimony) in tobacco smoke [30].

There are inverse relations between smoking and levels of important nutrients, independent of dietary intake [31]. We observed that smokers had significantly lower cobalt and molybdenum (adjusted) than nonsmokers. Our findings are similar to those reported for current vs. former or never smokers [32]. While human cobalt deficiencies have not been reported, cobalt is a component of vitamin B_12_ and smoking interferes with absorption of vitamin B_12_ [32,33]. Molybdenum is a co-factor for oxidoreductases such as xanthine oxidase [33]. Molybdenum-deficient soil has been considered a possible factor in regions with high rates of esophageal cancer [34,35]. An area of possible research is the relation between smoking, reduced levels of essential elements, and smoking-related morbidities and co-morbidities. Additional research is needed to determine if smokers have reduced cobalt and molybdenum due to dietary deficiencies or independent of other potentially confounding variables.

Our study is subject to several possible limitations. First, because our nonsmoker definition consisted of those that reported not smoking in the last five days, the upper ranges of SHS-exposed nonsmokers may include recent quitters and it is also possible that SHS-exposed nonsmokers included misidentified occasional smokers whose cotinine levels can overlap with SHS-exposed nonsmokers [36]. Dual characterization of nonsmoker status by self-report and serum cotinine eliminated nonsmokers with cotinine > 10 ng/mL. If the cotinine measurement condition is omitted from our nonsmoker definition, the estimated number of nonsmokers would increase by only 1%.

Other potential confounders not considered in our analyses are dietary sources of metals, hobbies involving metals, lead paint in the home, urban residence, or occupation. Information on some of these potential confounders (e.g., hobbies involving metals) is not available for the NHANES population. Compared with workers in some industries, however, the prevalence of elevated exposures to metals such as lead, cadmium, or antimony in the general population is low and occupational exposure in the NHANES population is expected to be rare [7]. Consequently, an assumption in our study is that while these potential confounders may differentially impact population groups—in particular those of lower socioeconomic status—for lead, the impact is likely minimal given the few variations observed in comparisons of those above and below the poverty threshold. Confounding from other potential sources of metal exposure should be carefully addressed in studies concerned with causality.

The representative nature of the data was an important strength as this enables some generalizability of the results. For example, we observed higher adjusted cadmium for females for all exposure groups and, like others [21], also higher levels in females in the overall sample. Additionally, including 6 years of data, including the over-sampling of Mexican Americans and non-Hispanic blacks, allowed adequate power for race/ethnicity comparisons. We found racial/ethnic differences in cadmium and lead. For example, non-Hispanic white nonsmokers_low_ had higher adjusted cadmium than did non-Hispanic blacks or Mexican Americans. Also, nonsmoking Mexican Americans with no or low SHS exposure had higher lead than non-Hispanic whites. Non-Hispanic blacks had the highest unadjusted lead in the overall sample, amongst nonsmokers_high_, and smokers. After adjustment, the differences were no longer significant. This observation may be explained by the significantly greater creatinine concentrations among non-Hispanic blacks than non-Hispanic whites or Mexican Americans [37]. Adjusting for creatinine content resulted in differences across exposure groups becoming significant for 6 metals: mercury, beryllium, cesium, cobalt, platinum, and thallium. Presenting both adjusted and unadjusted concentrations illustrates the importance of considering the appropriateness of creatinine correction based on the study population and the research questions.

Blood lead measurements are preferred to evaluate lead exposure [21]. Thus, a second limitation of the study is that urine lead measurements are more variable than blood levels [21]. It is possible that blood lead with less intra-individual variability may show different patterns in smoke-exposed individuals. In contrast, urine cadmium is a more reliable measure of chronic cadmium exposure than blood cadmium [7].

Although our study addresses 12 metals previously reported in tobacco smoke, it is not an exhaustive analysis of all metals in tobacco smoke. Data for several toxicologically-important chemicals (e.g., chromium, nickel, and arsenic) are not available for all waves of NHANES data in this study. Additionally, NHANES urine metals data are not available for children younger than 6 years. Finally, the study does not consider co-morbidities such as decreased renal function.

For some metals (e.g., mercury and thallium), our data suggest that tobacco smoke is not a significant source of exposure because levels were higher among nonsmokers than smokers. Counterfeit cigarettes can contain higher levels of toxic metals (e.g., cadmium, lead, and thallium) than legal cigarettes [38]. Consequently, consumption of counterfeit cigarettes or cigarettes with tobacco grown in sludge amended soil may exacerbate the problem [39].

## 3. Experimental Section

All data were obtained from the NHANES series conducted by the Centers for Disease Control and Prevention’s (CDC’s) National Center for Health Statistics (http://www.cdc.gov/nchs/nhanes.htm). NHANES is representative of civilian, noninstitutionalized residents of the United States 2 months or older. Three waves of NHANES data were included: 1999–2000, 2001–2002, and 2003–2004. The analysis file was limited to respondents who completed the health examination component of the survey, those with serum cotinine, urine creatinine, and the urine metals antimony, barium, beryllium, cadmium, cesium, cobalt, lead, mercury, molybdenum, platinum, thallium, and tungsten measurements, and those that responded to tobacco questions. Urine metals were measured for participants 6 years or older. The tobacco questions were asked of respondents 12 years or older.

**Figure 1.**
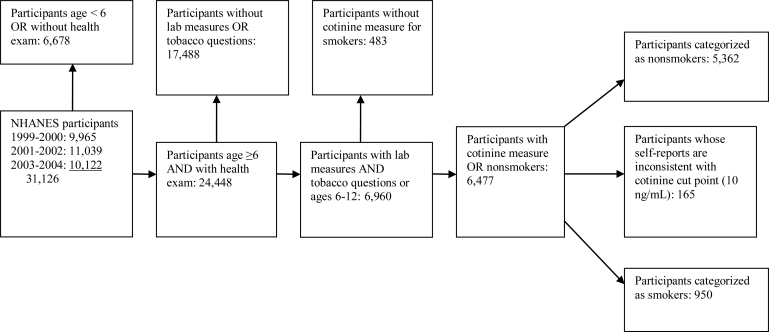
Analytic sample.

The final analysis file contained 6,312 respondents with the laboratory measurements, smoker or nonsmoker status based on responses to the tobacco questions and serum cotinine levels (further described below), and a valid (nonzero) weight variable. The development of the analysis file is presented in Figure 1.

Respondents 12 years or older who answered “No” to the question, “During the past 5 days, did you use any product containing nicotine including cigarettes, pipes, cigars, chewing tobacco, snuff, nicotine patches, nicotine gum, or any other product containing nicotine” were considered nontobacco users. Any participant younger than 12 years was considered a nontobacco user, but was excluded if their cotinine was ≥ 10 ng/ml because nonsmokers in NHANES have been defined by others [40] as persons with serum cotinine < 10 ng/mL. Self-reported nontobacco users 12 years or older with a cotinine measurement ≥ 10 ng/mL were also excluded from the analyses. A nonsmoker was defined as a self-reported nontobacco user with a cotinine measurement < 10 ng/mL. Nineteen self-reported nonsmokers in the final analytic sample had cotinine levels above 10 ng/mL and were excluded.

Smokers were defined as respondents 12 years or older whose only direct source of nicotine exposure was cigarettes. Smokers were those who answered “Yes” to the question, “During the past 5 days, did you use any product containing nicotine including cigarettes, pipes, cigars, chewing tobacco, snuff, nicotine patches, nicotine gum, or any other product containing nicotine” and did not use non-cigarette sources of nicotine as indicated by answering “No” to additional questions asking if they smoked pipes, cigars, used chewing tobacco, snuff or other nicotine products in the past 5 days. Smokers that used other non-cigarette sources of nicotine were excluded.

For cotinine levels below the level of detection, we used an estimated value of 0.035 ng/mL (i.e., level of detection, 0.050 ng/mL, divided by the square root of 2) when calculating geometric mean urine levels following procedures outlined in previous research [41]. Due to the highly skewed nature of cotinine levels in the study population [40], the geometric mean of cotinine, 0.256 ng/mL, was chosen as the cutoff point to define low and high SHS exposures. Exposure was categorized into four levels: self-identified, unexposed nonsmokers (nonsmokers) — nonsmokers with cotinine ≤ 0.035 ng/mL; nonsmokers with low SHS exposure (nonsmokers_low_) — nonsmokers with cotinine > 0.035 ng/mL and ≤ 0.256 ng/mL; nonsmokers with high SHS exposure (nonsmokers_high_) — nonsmokers with cotinine > 0.256 ng/mL and ≤ 10 ng/mL; and smokers—self-identified cigarette smokers with cotinine > 10 ng/mL. 146 self-reported smokers in the final analytic sample had cotinine levels below 10 ng/mL.

Participants who described themselves as “non-Hispanic white,” “non-Hispanic black,” and “Mexican American” were assessed and findings for these racial/ethnic subgroups are presented. “Other race/ethnicity,” were included in total population estimates but were not presented due to the small number of participants in this category. Participant age was categorized into 6 groups: 6–11, 12–18, 19–34, 35–49, 50–64, and 65 years and older.

The poverty-to-income ratio (PIR)—the ratio between family income and the poverty threshold, based on income thresholds that vary by family size and composition that are updated annually for inflation with the Consumer Price Index (U.S. Census Bureau, 2003, 2007) — was used to create a dichotomous variable to indicate if the participant was above (> 1.00) or below the poverty threshold (< 1.00). The poverty threshold was used as an indicator of socioeconomic status (SES). As reported in previous NHANES research [42], poverty is a useful indicator of SES because, unlike other indicators of SES like education or occupation, it provides a comparable measure of SES across a broad range of ages.

Biological samples (blood and urine) were collected during a standardized physical examination conducted in a mobile examination center (MEC) and stored cold or frozen until laboratory analyses were conducted. Serum cotinine was measured by a high-performance liquid chromatography/atmospheric-pressure ionization tandem mass spectrometry (LC/MS/MS) method that has been described previously [43]. Urine metals were measured by inductively coupled plasma-mass spectrometry as described previously [44]. Urine creatinine was measured using an automated colorimetric determination based on a modified Jaffe reaction [37]. Further descriptions of sample collection and laboratory methods for cotinine, creatinine, and the metals are available at: http://www.cdc.gov/nchs/nhanes.htm. Two sets of urine metal measures were created. One is the metal concentration adjusted for the creatinine content of the urine (μg/g creatinine) (adjusted). Creatinine correction adjusts for urine dilution and is typically performed with spot urine samples [37]. The other is the unadjusted urine metal concentration (μg/L) (unadjusted). Creatinine correction is commonly used in homogeneous populations; however, multiple demographic groups, such as in this study, increase the variability in creatinine levels [37,45]. Cadmium levels in the 1999–2002 data were corrected for molybdenum oxide interference. The correction resulted in corrected values less than zero being assigned a value of zero (http://www.cdc.gov/nchs/data/nhanes/frequency/lab06hm_doc.pdf). There were 49 cases with zero values in the final analytic sample. All zero values were set to missing in the analysis file to insure proper generation of the geometric mean.

We calculated estimates using the sub-sampling weights to represent nonsmokers ≥ 6 years and smokers ≥ 12 years in the United States. In 1999–2002, urine mercury was only measured for females 16–49 years. Starting in 2003, urine mercury was measured for males and females ≥ 6 years. For consistency across all 3 waves of data, the mercury analysis only included data for females 16–49 years.

Sampling weights provide population estimates that adjust for unequal probabilities of selection and account for nonresponses. The weights were post-stratified to the U.S. population as estimated by the Census Bureau. For use with multi-wave data, we calculated analytic survey weight following the NHANES documentation provided on their website. Specifically, this involved taking 2/3 of the special four-year MEC weight for 1999–2002 and 1/3 of the two-year MEC weight for 2003–2004. For analyses, we used SAS (SAS Institute, Inc., Cary, NC) and SUDAAN (Research Triangle Institute, Research Triangle Park, NC)—a program that adjusts for complex sample design when variance estimates are calculated.

Estimates for the geometric mean, with 95% confidence intervals, were calculated by demographic characteristic and for each exposure level. A linear regression analysis was then used to identify metals whose geometric mean is significantly different between the exposure groups, with log transformations of adjusted or unadjusted metal concentrations as the dependent variable and exposure group as the independent variable. If an overall difference was significant between the groups, t-tests were performed to identify the specific difference(s) for exposure group pairs. T-test comparisons for participants 12 years or older were “nonsmoker vs. nonsmoker_low_”, “nonsmoker_low_ vs. nonsmoker_high_”, “nonsmoker_high_ vs. smoker” and “nonsmoker vs. smoker”. For children 6–11 years, t-test comparisons were “nonsmoker vs. nonsmoker_low_”, “nonsmoker_low_ vs. nonsmoker_high_” and “nonsmoker vs. nonsmoker_high_”. Additional analyses were performed to examine differences in cadmium or lead levels across race/ethnicity and between genders. The comparison reference group for race/ethnicity is non-Hispanic white and for gender is male. Bonferroni correction was used to control the type I errors on multiple comparisons.

## 4. Conclusions

The U.S. Surgeon General’s recent declaration of no risk-free level of SHS exposure [1] underscores the importance of characterizing active and passive exposures to harmful tobacco smoke constituents. Some chemicals, notably cadmium and lead, will accumulate in the body. Our findings show SHS-exposed children, a population particularly vulnerable to the toxic effects of lead at low levels of exposure [15,16] have higher levels of urine lead than children without SHS exposure. Urine lead levels respond rapidly to changes in body lead and increase with increasing lead exposure [46] and our findings suggest the need for confirmatory study of blood lead levels among SHS exposed youth. Older smokers in our study had cadmium levels high enough to raise concerns that they are at risk for cadmium-related toxicity. Thus, our findings indicate that active and passive smoking should be considered in future investigations to ascertain the role of metals in the disease process. This finding is especially relevant for some minorities and socio-economically disadvantaged groups [47].

## Figures and Tables

**Table 1.** Sample Size by Demographic Characteristics for 12 Urinary Metals.

Demographic Characterisitics	Cd	Pb	Hg	Sb	Ba	Be	Cs	Co	Mo	Pt	Tl	W
Total	6,043	6,270	1,422	6,110	6,031	6,270	6,270	6,270	6,102	6,270	6,200	6,137
AGE(years)												
2:[6–12)	776	834	n/a^a^	815	796	834	834	834	807	834	824	813
3:[12–19)	1,461	1,523	297	1,485	1,450	1,523	1,523	1,523	1,477	1,523	1,499	1,483
4:[19–35)	1,077	1,103	626	1,074	1,071	1,103	1,103	1,103	1,084	1,103	1,091	1,079
5:[35–50)	918	939	499	912	913	939	939	939	919	939	938	925
6:[50–65)	800	822	n/a	797	786	822	822	822	802	822	811	803
7:[65+)	1,011	1,049	n/a	1,027	1,015	1,049	1,049	1,049	1,013	1,049	1,037	1,034
RACE/ETHNICITY												
Non-Hispanic White	2,453	2,532	583	2,485	2,480	2,532	2,532	2,532	2,477	2,532	2,500	2,502
Non-Hispanic Black	1,477	1,511	329	1,498	1,488	1,511	1,511	1,511	1,493	1,511	1,498	1,492
Mexican American	1,712	1,809	400	1,728	1,649	1,809	1,809	1,809	1,729	1,809	1,786	1,730
GENDER												
Male	2,937	3,052	n/a	2,971	2,933	3,052	3,052	3,052	2,966	3,052	3,016	2,978
Female	3,106	3,218	1,422	3,139	3,098	3,218	3,218	3,218	3,136	3,218	3,184	3,159
POVERTY INDEX												
Below poverty index	1,339	1,403	352	1,369	1,350	1,403	1,403	1,403	1,354	1,403	1,381	1,381
Above poverty index	4,220	4,360	967	4,257	4,215	4,360	4,360	4,360	4,259	4,360	4,320	4,269

^a^ Data not available for this group.

**Table 2.** Geometric Means and P-values Overall and by Level of Smoke Exposure for 12 Urinary Metals.

Metal	Creatinine Adjustment	Overall Geometric Mean metal level	Nonsmokers			Smokers	P-value^a^
			Geometric Mean metal level without SHS	Geometric Mean metal level with Low SHS	Geometric Mean metal level with High SHS	Geometric Mean metal level	
Cadmium	Adjusted^b^	0.22 (0.21,0.23)	0.21 (0.20,0.22)	0.20 (0.18,0.21)^e^	0.16 (0.14,0.17)^f^	0.34 (0.30,0.37)^f^	0.0000
Cadmium	Unadjusted^c^	0.22 (0.21,0.23)	0.20 (0.19,0.21)	0.21 (0.19,0.23)^d^	0.17 (0.15,0.19)^f^	0.37 (0.34,0.41)^f^	0.0000
Lead	Adjusted	0.66 (0.64,0.68)	0.61 (0.58,0.63)^d^	0.67 (0.64,0.70)	0.68 (0.62,0.73)	0.73 (0.69,0.78)^f^	0.0001
Lead	Unadjusted	0.67 (0.65,0.70)	0.58 (0.55,0.60)^f^	0.69 (0.64,0.75)	0.74 (0.66,0.82)	0.82 (0.76,0.89)^f^	0.0000
Mercury	Adjusted	0.65 (0.60,0.71)	0.75 (0.66,0.85)^d^	0.60 (0.53,0.67)	0.62 (0.46,0.77)	0.57 (0.51,0.63)^e^	0.0053
Mercury	Unadjusted	0.64 (0.57,0.72)	0.67 (0.57,0.78)	0.59 (0.47,0.71)	0.77 (0.56,0.98)	0.58 (0.48,0.67)	
Antimony	Adjusted	0.11 (0.10,0.12)	0.11 (0.10,0.11)	0.11 (0.10,0.12)	0.11 (0.10,0.12)	0.11 (0.10,0.12)	
Antimony	Unadjusted	0.11 (0.11,0.12)	0.10 (0.10,0.11)^e^	0.11 (0.11,0.12)	0.12 (0.11,0.13)	0.13 (0.12,0.14)^f^	0.0000
Barium	Adjusted	1.43 (1.36,1.50)	1.42 (1.34,1.50)	1.43 (1.32,1.55)	1.47 (1.30,1.63)	1.42 (1.31,1.54)	
Barium	Unadjusted	1.47 (1.39,1.55)	1.34 (1.26,1.43)	1.50 (1.36,1.64)	1.60 (1.39,1.81)	1.60 (1.44,1.75)^e^	0.0019
Beryllium	Adjusted	0.09 (0.08,0.09)	0.09 (0.09,0.10)^e^	0.08 (0.08,0.09)	0.08 (0.07,0.09)	0.08 (0.07,0.08)^f^	0.0000
Beryllium	Unadjusted	0.09 (0.09,0.09)	0.09 (0.09,0.09)	0.09 (0.09,0.09)	0.09 (0.09,0.09)	0.09 (0.09,0.09)	
Cesium	Adjusted	4.47 (4.35,4.59)	4.86 (4.67,5.05)^e^	4.42 (4.25,4.59)^d^	4.07 (3.85,4.28)	4.08 (3.93,4.24)^f^	0.0000
Cesium	Unadjusted	4.57 (4.37,4.77)	4.60 (4.34,4.87)	4.60 (4.25,4.95)	4.45 (4.07,4.82)	4.57 (4.28,4.87)	
Cobalt	Adjusted	0.34 (0.33,0.35)	0.35 (0.33,0.37)	0.34 (0.32,0.35)	0.35 (0.33,0.38)^e^	0.32 (0.29,0.34)^d^	0.0063
Cobalt	Unadjusted	0.35 (0.33,0.37)	0.33 (0.31,0.35)	0.35 (0.32,0.38)	0.39 (0.35,0.42)	0.35 (0.32,0.38)	0.0246
Molybdenum	Adjusted	41.69(40.19,43.18)	46.33(44.20,48.47)	42.79(40.29,45.29)	41.89(38.86,44.93)^d^	32.67(30.77,34.56)^d^	0.0000
Molybdenum	Unadjusted	42.71(40.66,44.76)	43.92(41.35,46.49)	44.63(40.48,48.78)	45.83(40.94,50.72)^d^	36.53(33.25,39.81)^d^	0.0032
Platinum	Adjusted	0.03 (0.03,0.04)	0.04 (0.04,0.04)	0.03 (0.03,0.04)	0.03 (0.03,0.03)	0.03 (0.03,0.03)^e^	0.0027
Platinum	Unadjusted	0.04 (0.03,0.04)	0.04 (0.03,0.04)	0.04 (0.03,0.04)	0.04 (0.03,0.04)	0.04 (0.03,0.04)	
Thallium	Adjusted	0.16 (0.16,0.16)	0.17 (0.16,0.17)	0.16 (0.16,0.17)	0.16 (0.15,0.17)^f^	0.14 (0.13,0.14)^f^	0.0000
Thallium	Unadjusted	0.16 (0.16,0.17)	0.16 (0.15,0.17)	0.17 (0.16,0.18)	0.17 (0.16,0.19)	0.15 (0.14,0.16)	
Tungsten	Adjusted	0.08 (0.07,0.08)	0.08 (0.07,0.08)	0.08 (0.07,0.08)	0.08 (0.07,0.09)	0.07 (0.06,0.08)	
Tungsten	Unadjusted	0.08 (0.07,0.08)	0.07 (0.07,0.08)	0.08 (0.07,0.09)	0.09 (0.08,0.10)	0.08 (0.07,0.09)	0.0240

^a^ A statistically significant difference in log transformed mean levels between the exposure groups (nonsmoker, nonsmoker_low_, nonsmoker_high_, smoker) determined by linear regression.

^b^ Units are μg/g creatinine.

^c^ Units are μg/L.

For statistical analysis of differences in mean urine metal levels the following t-tests comparisons were performed: nonsmokers and nonsmokers_low_; nonsmokers_low_ and nonsmokers_high_; nonsmokers_high_ and smokers; smokers and nonsmokers;

^d^ p-value less than 0.05;

^e^ p-value less than 0.01;

^f^ p-value less than 0.001.

**Table 3.** Geometric Means and P-values Overall and by Level of Smoke Exposure and by Demographic Characteristics for Cadmium (Cd).

Demographic Characteristics	Creatinine Adjustment	Overall Geometric Mean Cd level	Nonsmokers			Smokers	P-value^a^
			Geometric Mean Cd level without SHS	Geometric Mean Cd level with Low SHS	Geometric Mean Cd level with High SHS	Geometric Mean Cd level	
AGE(years)							
2:[6–12)^b^	Adjusted^c^	0.09 (0.08,0.10)	0.08 (0.07,0.10)	0.09 (0.07,0.10)	0.09 (0.08,0.11)	n/a^d^	
	Unadjusted^e^	0.08 (0.07,0.09)	0.08 (0.06,0.09)	0.08 (0.06,0.10)	0.08 (0.07,0.10)	n/a	
3:[12–19)	Adjusted	0.09 (0.08,0.09)	0.09 (0.08,0.10)	0.09 (0.08,0.10)	0.08 (0.07,0.09)	0.08 (0.07,0.10)	
	Unadjusted	0.12 (0.11,0.13)	0.11 (0.10,0.13)	0.13 (0.11,0.15)	0.12 (0.11,0.14)	0.12 (0.09,0.15)	
4:[19–35)	Adjusted	0.14 (0.13,0.15)	0.14 (0.13,0.15)	0.11 (0.09,0.13)	0.13 (0.11,0.15)	0.18(0.16,0.20)^f^	0.0005
	Unadjusted	0.18 (0.17,0.20)	0.16 (0.14,0.18)	0.14 (0.10,0.17)	0.19 (0.15,0.22)	0.24(0.20,0.27)^h^	0.0002
5:[35–50)	Adjusted	0.27 (0.25,0.29)	0.21 (0.19,0.23)	0.24 (0.20,0.27)	0.22(0.18,0.26)^h^	0.45(0.40,0.50)^h^	0.0000
	Unadjusted	0.28 (0.25,0.30)	0.21 (0.18,0.24)	0.26 (0.22,0.29)	0.22(0.17,0.27)^h^	0.45(0.39,0.50)^h^	0.0000
6:[50–65)	Adjusted	0.40 (0.37,0.43)	0.33 (0.30,0.37)	0.38 (0.31,0.45)	0.36(0.30,0.42)^h^	0.67(0.58,0.76)^h^	0.0000
	Unadjusted	0.35 (0.32,0.39)	0.28 (0.25,0.32)	0.34 (0.27,0.41)	0.30(0.19,0.41)^h^	0.65(0.54,0.76)^h^	0.0000
7:[65+)	Adjusted	0.46 (0.43,0.49)	0.43 (0.39,0.46)	0.40(0.36,0.43)^g^	0.54(0.46,0.63)^h^	1.04(0.90,1.18)^h^	0.0000
	Unadjusted	0.36 (0.33,0.39)	0.31 (0.28,0.35)	0.35 (0.29,0.40)	0.43(0.34,0.51)^h^	0.90(0.72,1.08)^h^	0.0000
RACE/ETHNICITY							
Non-Hispanic White	Adjusted	0.23 (0.21,0.24)	0.22 (0.20,0.23)	0.21(0.18,0.24)^g^	0.15(0.14,0.17)^h^	0.34(0.30,0.38)^h^	0.0000
	Unadjusted	0.22 (0.20,0.23)	0.20 (0.18,0.21)	0.20(0.18,0.23)^f^	0.16(0.14,0.18)^h^	0.34(0.30,0.38)^h^	0.0000
Non-Hispanic Black	Adjusted	0.20 (0.18,0.21)^j^	0.19 (0.17,0.22)	0.16 (0.14,0.18)^i^	0.15 (0.13,0.17)^h^	0.36 (0.30,0.42)^h^	0.0000
	Unadjusted	0.29 (0.26,0.32)^k^	0.27 (0.23,0.32)^j^	0.23 (0.20,0.26)	0.21 (0.18,0.24)^h^,^j^	0.60 (0.50,0.70)^h^,^k^	0.0000
Mexican American	Adjusted	0.18 (0.16,0.19)^k^	0.17 (0.16,0.19)^k^	0.16 (0.14,0.18)^j^	0.15 (0.12,0.17)^h^	0.28 (0.23,0.34)^h^	0.0000
	Unadjusted	0.19 (0.18,0.21)^i^	0.17 (0.16,0.19)^i^	0.18 (0.16,0.21)	0.16 (0.13,0.19)^h^	0.36 (0.30,0.41)^h^	0.0000
GENDER							
Male	Adjusted	0.18 (0.17,0.19)	0.16 (0.15,0.17)	0.16 (0.14,0.18)	0.13 (0.12,0.15)^h^	0.29 (0.25,0.32)^h^	0.0000
	Unadjusted	0.22 (0.21,0.23)	0.19 (0.18,0.21)	0.20 (0.17,0.23)	0.16 (0.13,0.18)^h^	0.38 (0.33,0.43)^h^	0.0000
Female	Adjusted	0.26 (0.25,0.28)^k^	0.26 (0.24,0.27)^k^	0.25 (0.22,0.28)^h^,^k^	0.18 (0.16,0.20)^h^,^k^	0.40 (0.35,0.45)^h^,^k^	0.0000
	Unadjusted	0.23 (0.21,0.24)	0.20 (0.19,0.22)	0.21 (0.19,0.24)	0.19 (0.16,0.21)^h^	0.37 (0.32,0.42)^h^	0.0000
POVERTY INDEX							
Below poverty index	Adjusted	0.20 (0.18,0.21)	0.19 (0.17,0.22)	0.17 (0.14,0.20)	0.14 (0.12,0.16)^h^	0.31 (0.27,0.36)^h^	0.0000
	Unadjusted	0.23 (0.20,0.25)	0.19 (0.16,0.22)	0.21 (0.17,0.25)	0.16 (0.14,0.18)^h^	0.40 (0.33,0.47)^h^	0.0000
Above poverty index	Adjusted	0.22 (0.21,0.23)	0.21 (0.20,0.22)	0.20 (0.18,0.22)^g^	0.16 (0.14,0.17)^h^	0.34 (0.30,0.38)^h^	0.0000
	Unadjusted	0.22 (0.21,0.23)	0.20 (0.18,0.21)	0.20 (0.18,0.23)	0.17 (0.15,0.19)^h^	0.37 (0.32,0.42)^h^	0.0000

^a^ A statistically significant difference in log transformed mean levels between the exposure groups (nonsmoker, nonsmoker_low_, nonsmoker_high_, smoker) determined by linear regression.

^b^ For age categories, a square bracket indicates inclusion of the interval end point, and a parenthesis indicates exclusion.

^c^ Units are μg/g creatinine.

^d^ Not applicable.

^e^ Units are μg/L.

For statistical analysis of differences in mean urine metal levels the following t-tests comparisons were performed: nonsmokers and nonsmokers_low_; nonsmokers_low_ and nonsmokers_high_; nonsmokers_high_ and smokers; smokers and nonsmokers; and nonsmoker vs. nonsmoker_high_ for children aged 6–11 years;

^f^ p-value less than 0.05;

^g^ p-value less than 0.01;

^h^ p-value less than 0.001. For statistical analysis of differences in mean urine cadmium levels across group comparisons, the following t-test comparisons were performed: non-Hispanic black versus non-Hispanic white, Mexican American versus non-Hispanic white, and female versus male;

^i^ p-value less than 0.05;

^j^ p-value less than 0.01;

^k^ p-value less than 0.001.

**Table 4.** Geometric Means and P-values Overall and by Level of Smoke Exposure and Demographic Characteristics for Lead (Pb).

Heavy Metal	Creatinine Adjustment	Overall Geometric Mean Pb level	Nonsmokers			Smokers	P-value^a^
			Geometric Mean Pb level without SHS	Geometric Mean Pb level with Low SHS	Geometric Mean Pb level with High SHS	Geometric Mean Pb level	
AGE (years)							
2:[6–12)^b^	Adjusted^c^	0.97 (0.90,1.03)	0.85 (0.77,0.93)	0.89(0.80,0.99)^g^	1.17(1.03,1.30)^h^	n/a^d^	0.0005
	Unadjusted^e^	0.87 (0.79,0.94)	0.78 (0.69,0.87)	0.79 (0.67,0.91)	1.04(0.87,1.20)^f^	n/a	0.0153
3:[12–19)	Adjusted	0.43 (0.41,0.44)	0.39(0.36,0.42)^f^	0.48 (0.43,0.53)	0.44 (0.39,0.49)	0.42 (0.38,0.46)	0.0390
	Unadjusted	0.59 (0.55,0.62)	0.49(0.45,0.54)^g^	0.67 (0.58,0.77)	0.66 (0.58,0.75)	0.63(0.53,0.73)^f^	0.0008
4:[19–35)	Adjusted	0.48 (0.45,0.51)	0.42 (0.38,0.46)	0.48 (0.43,0.52)	0.47 (0.40,0.53)	0.56(0.50,0.61)^h^	0.0005
	Unadjusted	0.61 (0.56,0.65)	0.48 (0.42,0.54)	0.59 (0.52,0.67)	0.66 (0.52,0.79)	0.76(0.65,0.87)^h^	0.0001
5:[ 35–50)	Adjusted	0.65 (0.61,0.69)	0.56 (0.51,0.61)	0.59 (0.53,0.65)	0.64(0.54,0.74)^g^	0.84(0.75,0.94)^h^	0.0000
	Unadjusted	0.66 (0.61,0.71)	0.55 (0.50,0.60)	0.64 (0.52,0.77)	0.65 (0.52,0.79)	0.84(0.75,0.93)^h^	0.0000
6:[50–65)	Adjusted	0.80 (0.75,0.85)	0.72 (0.67,0.78)	0.83 (0.75,0.91)	0.81 (0.67,0.95)	0.96(0.85,1.08)^h^	0.0002
	Unadjusted	0.71 (0.64,0.78)	0.62 (0.55,0.68)	0.74 (0.64,0.85)	0.67 (0.42,0.92)	0.94(0.78,1.10)^h^	0.0001
7:[65+)	Adjusted	0.91 (0.87,0.95)	0.88 (0.83,0.93)	0.92 (0.84,1.00)	0.99 (0.80,1.18)	1.11(0.96,1.26)^f^	0.0318
	Unadjusted	0.72 (0.66,0.77)	0.64(0.59,0.70)^f^	0.81 (0.70,0.92)	0.79 (0.62,0.97)	0.98(0.82,1.15)^h^	0.0008
RACE/ETHNICITY							
Non-Hispanic White	Adjusted	0.64 (0.62,0.66)	0.59(0.56,0.63)^f^	0.66 (0.62,0.69)	0.63 (0.57,0.70)	0.71(0.66,0.76)^g^	0.0011
	Unadjusted	0.61 (0.58,0.64)	0.54(0.51,0.57)^f^	0.64 (0.58,0.70)	0.65 (0.55,0.74)	0.72(0.65,0.78)^h^	0.0001
Non-Hispanic Black	Adjusted	0.66 (0.61,0.71)	0.55 (0.50,0.59)	0.59(0.54,0.64)^h^	0.73 (0.64,0.82)	0.82(0.71,0.92)^h^	0.0000
	Unadjusted	0.96 (0.89,1.03)^k^	0.76 (0.69,0.83)^k^	0.83(0.72,0.94)^f^,^j^	1.04(0.92,1.16)^f^,^k^	1.34(1.19,1.50)^h^,^k^	0.0000
Mexican American	Adjusted	0.81 (0.77,0.86)^k^	0.79 (0.75,0.83)^k^	0.83 (0.76,0.91)^k^	0.79 (0.64,0.93)	0.89 (0.75,1.03)^i^	
	Unadjusted	0.87 (0.81,0.92)^k^	0.79 (0.72,0.85)^k^	0.94 (0.82,1.06)^k^	0.87 (0.76,0.97)^j^	1.13(0.95,1.30)^h^,^k^	0.0002
GENDER							
Male	Adjusted	0.65 (0.62,0.67)	0.56(0.53,0.59)^h^	0.70 (0.65,0.76)	0.67 (0.59,0.74)	0.73(0.68,0.77)^h^	0.0000
	Unadjusted	0.80 (0.76,0.84)	0.69(0.64,0.73)^h^	0.87 (0.78,0.96)	0.78 (0.65,0.92)	0.95(0.86,1.04)^h^	0.0000
Female	Adjusted	0.67 (0.64,0.69)	0.65 (0.61,0.68)^k^	0.63 (0.60,0.67)^i^	0.69 (0.62,0.75)	0.74 (0.67,0.82)	0.0494
	Unadjusted	0.58 (0.55,0.60)^k^	0.51 (0.48,0.54)^k^	0.56(0.50,0.61)^f^,^k^	0.70 (0.61,0.79)	0.70(0.62,0.77)^h^,^k^	0.0000
POVERTY INDEX							
Below poverty index	Adjusted	0.74 (0.68,0.79)	0.70 (0.62,0.78)	0.70 (0.61,0.78)	0.81 (0.69,0.94)	0.73 (0.64,0.83)	
	Unadjusted	0.84 (0.78,0.91)	0.68 (0.59,0.77)	0.83 (0.72,0.95)	0.95 (0.81,1.09)	0.93(0.80,1.07)^f^	0.0015
Above poverty index	Adjusted	0.63 (0.61,0.65)	0.59(0.56,0.62)^f^	0.65 (0.61,0.68)	0.64 (0.58,0.69)	0.72(0.66,0.77)^h^	0.0007
	Unadjusted	0.63 (0.61,0.66)	0.56(0.53,0.59)^g^	0.65 (0.60,0.71)	0.68(0.60,0.77)^f^	0.78(0.70,0.86)^h^	0.0000

^a^ A statistically significant difference in log transformed mean levels between the exposure groups (nonsmoker, nonsmoker_low_, nonsmoker_high_, smoker) determined by linear regression.

^b^ For age categories, a square bracket indicates inclusion of the interval end point, and a parenthesis indicates exclusion.

^c^ Units are μg/g creatinine.

^d^ Not applicable.

^e^ Units are μg/L.

For statistical analysis of differences in mean urine metal levels the following t-tests comparisons were performed: nonsmokers and nonsmokers_low_; nonsmokers_low_ and nonsmokers_high_; nonsmokers_high_ and smokers; smokers and nonsmokers; and nonsmoker vs. nonsmoker_high_ for children aged 6–11 years.

^f^ p-value less than 0.05;

^g^ p-value less than 0.01;

^h^ p-value less than 0.001. For statistical analysis of differences in mean urine lead levels across group comparisons, the following t-test comparisons were performed: non-Hispanic black versus non-Hispanic white, Mexican American versus non-Hispanic white, and female versus male.

^i^ p-value less than 0.05;

^j^ p-value less than 0.01;

^k^ p-value less than 0.001.
